# The Milk of Syphilitic Nurses

**Published:** 1868-07

**Authors:** 


The Milk of Syphilitic Nurses.—Dr. Padova has inoculated this milk on some healthy individuals, and, as the result of the operation was negative, he hastily concludes that this fluid has no infectious properties; forgetting that the milk placed under the skin, and the same secretion obtained by suction, and reaching the stomachs of children, are widely different in their effects.—*Lancet.*

